# Prenatal exposure to cooking gas and respiratory health in infants is modified by tobacco smoke exposure and diet in the INMA birth cohort study

**DOI:** 10.1186/1476-069X-12-100

**Published:** 2013-12-01

**Authors:** Ana Esplugues, Marisa Estarlich, Jordi Sunyer, Virginia Fuentes-Leonarte, Mikel Basterrechea, Martine Vrijheid, Isolina Riaño, Loreto Santa-Marina, Adonina Tardón, David Martinez, Ferran Ballester

**Affiliations:** 1 CIBER Epidemiología y Salud Pública (CIBERESP), Melchor Fernández Almagro, 3-5, 28029, Madrid, Spain; 2 Center for Public Health Research (CSISP-FISABIO), Avda. de Catalunya, 21 / 46020, Valencia, Spain; 3 Faculty of Nursing and Chiropody, University of Valencia, Av. Blasco Ibáñez, 13, 46010 Valencia, Spain; 4 Center for Research in Environmental Epidemiology (CREAL), Doctor Aiguader, 88, E-08003 Barcelona, Spain; 5 Hospital del Mar Research Institute (IMIM), Doctor Aiguader, 88, 08003 Barcelona, Spain; 6 Pompeu Fabra University, Plaça de la Mercè, 10-12, 08002 Barcelona, Spain; 7 Public Health Division of Gipuzkoa, Avda. de Navarra, 4, 20013 Donostia-San Sebastián Basque Government, Spain; 8 Health Research Institute, BIODONOSTIA, Hospital Donostia, Pº Doctor Begiristain, s/n 20014, DonostiaBasque Country, Spain; 9 University of Oviedo, C/ González Besada, nº 13, Oviedo, Asturias, Spain; 10 Unit of Addictive Disorders, San Marcelino Primary Health Care Center, SAN PIO X, 32ac (C.S. San Marcelino) 46017 Valencia, Spain

**Keywords:** Gas cooking, Infant, Wheezing, Chestiness, Otitis, Pregnancy, Fruit and vegetable consumption, Tobacco

## Abstract

**Background:**

Studies that have evaluated the association between exposure to gas appliances emissions at home with respiratory health in children obtained heterogeneous and limited results. The aim of this study is to analyze the association between the use of gas cooking at home during pregnancy and respiratory problems in children during their first year of life.

**Methods:**

In the years 2003 through 2008 pregnant women were enrolled in 4 Spanish areas and visited in different age-points following a common protocol. Outcomes studied (from a questionnaire) were any episode of lower respiratory tract infection (LRTI), wheezing, persistent cough, chestiness and otitis. The association between exposure to gas cooking at home and respiratory outcomes was assessed using logistic regression and adjusting by confounding variables. Some potential effect modifiers (i.e. smoking, fruit and vegetables consumption) were examined.

**Results:**

Among the 2003 children included in the study, a total of 731 (36.6%) had a LRTI episode, 693 (34.6%) experienced wheezing, 302 (15.5%) a persistent cough, 939 (47.4%) chestiness and 620 (31.2%) had an episode of otitis during their first year of life. Gas cookers were present in 45.5% of homes. Exposure to gas cooking in homes was not associated with respiratory outcomes Odds Ratios (OR) were close to 1 and not statistically significant. However, a positive association was found for otitis among infants whose mothers reported low intakes of fruit and vegetables during pregnancy [OR (95% CI) = 1.38 (1.01-1.9)] and also wheezing and chestiness were associated with gas cookers among those children whose mothers smoked during pregnancy.

**Conclusions:**

In susceptible subjects (those whose mothers smoke and consumed below average fruit and vegetables) we found an association between exposure to gas cooking during pregnancy and risk of wheezing, chestiness and otitis during the first year of life. But more research is needed regarding not only gas cooking and respiratory health but also the possible effect modifier role of diet and tobacco.

## Introduction

Respiratory problems are a major cause of morbidity in young children worldwide. In developing countries the use of solid fuels for cooking and heating has been identified as a major risk to children's respiratory health and mortality. In developed countries, the use of solid fuels is no longer the most used (except in rural areas) as it was replaced by the use of gas or electrical appliances. Presence of gas cookers, gas heaters and other gas appliances at home are considered one of the main sources of indoor air pollutants [1]. Using these gas appliances a complex mix of by-products of gas combustion and other compounds, such as nitrogen dioxide (NO_2_) or ultra fine particles are emitted [2]. Based on the Hasselblad meta-analysis the WHO states that, having a gas stove was equivalent to an increased average indoor NO_2_ level of 28 μg/m^3^ compared to homes with electric stoves [1][3]. Furthermore, the use of gas cookers particularly in poorly ventilated spaces can experience peak nitrogen dioxide exposure in excess of WHO (2010) guidelines of indoor air quality. Accordingly a study with real-time sensors for identifying peak exposures has identified cooking with gas appliances as one of the main contributors to peak exposures of NO_2_ (levels as high as 1501 μg/m^3^) [4].

Respiratory system development and maturation commences in embryonic life and continues postnatally through to adolescence. Several factors, such as air pollutants, could interfere with the developmental program during any of the phases of development, and this may result in altered lung function and/or increased risk of disease in later life [5,6]. Moreover some factors, such as nutritional status or genetics, modulate the susceptibility of lungs to environmental pollutants [7,8].

Although a significant number of publications have evaluated the association between exposure to current levels of NO_2_ indoors or the presence of gas appliances with respiratory health in children [1,9-11], their results are heterogeneous and limited. Even in case of population-based studies results are inconsistent on the association between asthma and emissions from gas cooking [11]. In contrast recent studies that evaluate prenatal exposure to outdoor air pollution indicate that it may play a role in the development of the respiratory and immune systems and have an effect on respiratory health and allergic responses during early life and beyond [12-17].

In the context of a prospective multicenter birth cohort the aim of this study is to analyze the association between the presence of gas cooking at home (an indicator of indoor air pollution) during pregnancy and respiratory problems in children during their first year of life.

## Materials and methods

### Study population

This study is based on four population-based birth cohorts, Asturias, Gipuzkoa, Sabadell and Valencia, from the Spanish INMA study [Environment and Childhood]. In the years 2003 through 2008 pregnant women were enrolled during the first trimester of pregnancy and visited in different age-points following a common protocol [18]. Participation rates at each follow-point are reported elsewhere in detail. Participation in the cohort was somewhat selective in that women from higher educational levels were more likely to participate and to continue participation [18].

Outcomes, exposure and covariates were obtained from structured questionnaires administered by trained personnel during the first and third trimesters of pregnancy and first year of life. For children who continued in the study at 1 year (mean 14.5 months, 4.1 SD) of life we have complete information for 2235 children on respiratory outcomes and presence of gas cooking during pregnancy. Nevertheless to avoid recall bias we decided to remove from the analysis those children who completed the questionnaire after 30 months of life (39% of Asturias cohort). Finally 2003 children were included in the analysis (see Flowchart illustrating the main phases in the study Additional file 1). Informed consent was signed and the study was approved by the hospital ethics committees in the participating regions.

### Questionnaire data

Outcomes studied were any episode of LRTI (Lower Respiratory Tract Infection) diagnosed by a doctor (bronchitis, bronchiolitis or pneumonia), wheezing (defined as whistling sounds coming from the chest), persistent cough (more than three consecutive weeks), otitis and chestiness (noises or phlegm on the chest). All outcomes are referred to the child's first year of life period.

Exposure during pregnancy (asked at third trimester of pregnancy) was assessed taking into account the type of cooker (natural gas, butane gas, propane gas, electric, other), which was categorized into “gas cooker” (any type of gas cooker) and “no gas cooker” (electric and other).

Covariates considered were: age, sex, season of birth, preterm (<37 weeks of gestation) and small for gestational age (SGA, birth weight or length below the 10th percentile according to standard percentile charts for sex and gestational age in the Spanish population [19]), breastfeeding, parity, parental asthma, country of origin, maternal and paternal educational level, social class, furry pets, redecoration, active and passive smoking during pregnancy, ventilation, day care attendance, cleaning frequency, damp and type of residence zone (rural vs. urban). Social class was defined according to the highest status occupation of the mother during pregnancy (or of the father, if the mother did not work) using a widely used Spanish adaptation of the British classification system [20]. Class I + II included managerial workers, senior technical staff, and commercial managers; class III included skilled non-manual workers; and class IV + V, manual workers. Maternal diet was obtained at first trimester through a validated food frequency questionnaire and reported intakes were converted to estimate daily frequencies of fruit and vegetables [21].

### Statistical analysis

Logistic regression models tested for differences in respiratory outcomes between homes with and without a gas cooker. We calculated Odds Ratio (OR) and corresponding 95% confidence interval (CI), for the risk of LRTI, wheezing, cough, chestiness and otitis among children exposed prenatally to gas cooking. Models were adjusted for variables that were retained in the final model if they were related to the outcome (based on likelihood ratio (LR) tests with a p value of <0.10) or if the OR for the exposure of interest changed by ≥10% when they were excluded from the model. Cohort and child’s age were included in all models despite their statistical significance. We performed a combined analysis using meta-analysis techniques. Homogeneity among cohort estimates was quantified by means of the I-squared statistic (I^2^); when I^2^ was above 50%, the random-effect model was applied.

We then performed a sensitivity analysis in different subgroup populations such as SGA, preterm and those who did not change the type of cooking between pregnancy and the follow-up visit at first year. Besides that, and based on biological mechanisms, the effect modification of exposure to gas cooking association by smoking during pregnancy (tobacco smoke causes damage to lung defense mechanisms), and fruit and vegetable consumption (protecting the lung from oxidative damage) was assessed through stratified analysis and inclusion of the interaction terms in the models.

Analyses were carried out with Stata statistical package version 9.0 [22] and R 2.11.0 [23].

## Results

Of the 2003 children included in the analysis, a total of 731 (36.6%) had a LRTI episode, 693 (34.6%) experienced wheezing, 302 (15.5%) persistent cough, 939 (47.4%) chestiness and 620 (31.2%) otitis during the first year of life. Gas cookers were present in 45.5% of homes during pregnancy (912 subjects), although residents in Sabadell and Valencia were more exposed to gas cookers (61.2% and 64.5%, respectively) than the other two cohorts (Table 1).

**Table 1:** Cumulative incidence of respiratory infection and symptoms during the first year and use of gas cooker

	**Study population**									
	**Overall (n = 2003)**		**Asturias (n = 228)**		**Gipuzkoa (n = 527)**		**Sabadell (n = 544)**		**Valencia (n = 704)**	
Respiratory outcomes	N	%	N	%	N	%	N	%	N	%
LRTI	731	36.6	105	46.3	205	38.9	217	40.0	204	29.0
Wheezing	693	34.6	125	54.8	189	35.9	198	36.5	181	25.7
Cough	302	15.5	38	17.8	120	22.9	90	17.5	54	7.7
Otitis	620	31.2	81	36.2	189	36.0	178	33.2	172	24.4
Chestiness	939	47.4	149	68.3	175	33.5	319	59.1	296	42.2
Exposure to gas cooker	912	45.5	43	18.9	82	15.6	333	61.2	454	64.5

No statistically significant differences between children included in the analysis and those not included (n: 668) were observed in terms of mother’s country of origin, parity, active and passive smoking during pregnancy, type of residence zone, children’s sex, pets, damp and house ventilation (Additional file 2).

The relation of respiratory outcomes in the first year by individual and family characteristics is shown in additional file (see Additional file 3). The odds of all the outcomes were significantly higher in those children who attended day-care, boys (except for cough), and those whose parents had a history of allergies (except for cough and chestiness). Likewise, the OR of LRTI, wheezing and chestiness was higher in infants whose mother had higher parity and who smoked during pregnancy or was exposed to second hand smoke (wheezing, otitis and chestiness).

All results by cohort showed no significant heterogeneity (I^2^ < 50%). Therefore, a multivariate analysis with fixed effects was the method of analysis for the relationship between exposure variables and respiratory health outcomes. Exposure to gas cookers in the home was not associated with respiratory outcomes during the first year, i.e. all ORs were close to 1 and not statistically significant (Table 2). Several sensitivity analyses were performed, stratifying the analysis by potential effect modifiers (SGA, preterm, change in type of cooking) and the results remained stable (see Additional file 4).

**Table 2:** Association of exposure to gas cooker and respiratory problems during first year a birth cohort, Spain

	**Unadjusted model**			Adjusted model*		
	**OR**	**(95% CI)**		**OR**	**(95% CI)**	
LRTI	1.030	0.837	1.268	0.979	0.785	1.221
Wheezing	1.026	0.829	1.269	0.911	0.727	1.142
Cough	0.923	0.69	1.233	0.895	0.663	1.209
Chestiness	1.047	0.854	1.284	0.952	0.769	1.178
Otitis	1.090	0.879	1.354	1.068	0.856	1.332

*Adjusted variables: (LRTI) region, country of origin, parity, smoke during pregnancy, season of birth, parents allergic antecedents, sex, gestational age, cleaning frequency, day-care attendance, pets, child age at questionnaire administration; (WHEEZING) region, social class, parity, smoke during pregnancy, global passive tobacco exposure at pregnancy, parents allergic antecedents, sex, breastfeeding, day-care attendance, pets, child age at questionnaire administration ; (COUGH) region, social class, weeks of gestation, day-care attendance, child age at questionnaire administration; (CHESTINESS) region, mother's age, parity, smoke during pregnancy, sex, day-care attendance, pets, child age at questionnaire administration ; (OTITIS) region, educational level, home passive tobacco exposure at pregnancy, parents allergic antecedents, sex, day-care attendance, pets, child age at questionnaire administration.

When we stratified by smoking during pregnancy we found different results with two symptoms depending on prenatal smoking exposure (Table 3). Wheezing and chestiness showed some pattern of association with gas cookers among those children whose mothers smoked during pregnancy. On the other hand, when we stratified by fruit and vegetable intake (< 518 gr, median) we found a significant relationship between gas cookers and otitis among infants with low maternal intakes of fruit and vegetables during the first trimester of pregnancy.

**Table 3:** Associations between gas cooker and respiratory problems in several subsamples of INMA birth cohort Spain

	**Exposed to gas cooker**				
		**OR**	**(95% CI)**		**p-value interaction**
LRTI	Smoking during pregnancy				0.125
	No	0.943	0.739	1.203	
	Yes	1.093	0.631	1.894	
	Fruit and vegetable consumption				0.28
	< 518 g (median)	1.130	0.828	1.542	
	> 518 g (median)	0.835	0.605	1.152	
Wheezing					
	Smoking during pregnancy				0.002
	No	0.813	0.634	1.044	
	Yes	1.685	0.966	2.938	
	Fruit and vegetable consumption				0.899
	< 518 g (median)	0.951	0.694	1.304	
	> 518 g (median)	0.884	0.636	1.228	
Cough					
	Smoking during pregnancy				0.562
	No	0.950	0.684	1.320	
	Yes	0.641	0.297	1.386	
	Fruit and vegetable consumption				0.516
	< 518 g (median)	0.854	0.547	1.331	
	> 518 g (median)	0.933	0.616	1.414	
Chestiness					
	Smoking during pregnancy				0.036
	No	0.883	0.699	1.115	
	Yes	1.391	0.814	2.378	
	Fruit and vegetable consumption				0.753
	< 518 g (median)	0.996	0.741	1.338	
	> 518 g (median)	0.891	0.651	1.218	
Otitis					
	Smoking during pregnancy				0.062
	No	0.994	0.779	1.267	
	Yes	1.63	0.928	2.864	
	Fruit and vegetable consumption				0.022
	< 518 g (median)	1.389	1.013	1.905	
	> 518 g (median)	0.813	0.593	1.114	

## Discussion

In this birth cohort study carried out in several regions of Spain we did not find a relationship between cumulative incidence of respiratory outcomes during the 1^st^ year of life and gas cooker use during pregnancy. This finding is in parity with those of two birth cohorts, one in the United States of America (N = 936) [24] and the other in Sweden (N = 540) [25]. Both of them evaluated the respiratory effects (LRTI and recurrent wheezing) of gas appliances in small children and found no clear association. Additionally several studies that evaluate indoor exposure to NO_2_ with passive dosimeters in large cohorts observed similar findings [26,27].

On the other hand, typical exposure to gas from cookers is in the form of high peaks of pollutants, and in case of repeated exposure peaks, certain lung defence mechanisms may be overwhelmed, especially in subjects who are more vulnerable. Our results show that wheezing and chestiness were associated with gas cookers among those children whose mothers smoked during pregnancy. In the same way Sonnenschein-van der Voort et al. [28] observed an increased risk of wheezing in the first 3 years of life associated with higher levels of ambient PM_10_ and NO_2_ in those children exposed to tobacco smoke during fetal and infant life but not in those not exposed to tobacco smoke. Emenius and co-workers [25] when examining the role of indoor NO_2_, found no significant association with recurrent wheezing, but although indoor NO_2_ was not significant, they found a positive interaction between indoor NO_2_ and environmental tobacco smoke. Neuman et al. [29] in a pooled analysis of eight European birth cohorts found an independent effect of maternal smoking only during pregnancy on wheezing (OR 1.39 (95% CI: 1.08-1.77)) and asthma (OR: 1.65 (95% CI: 1.18-2.31)) in preschool children. Our results suggest that tobacco smoke exposure increases the vulnerability of the lungs to gas from cooking. The mechanism of health effects from these two exposures might be similar and would have life-long consequences in the development of the respiratory system. It is known that exposure to the components of tobacco smoke results in changes in the conducting airways and alveoli render the lungs of the offspring more susceptible to disease and reduced lung function [30].

Moreover our results show a higher risk of otitis from exposure to gas cookers among infants whose mothers consumed below average intake of fruit and vegetables during pregnancy. The association between traffic-related air pollution and otitis had been found before in prospective birth cohort studies [17,31]. And several studies suggest that dietary intake of fruit and vegetables might modify the adverse effect of air pollutants [7,8], also in children. This effect could be explained because a low antioxidant status in these children, influenced by low diet intakes of antioxidants (low intakes of fruit and vegetables), should lead or contribute to ear inflammation and infection. The antioxidant defense systems, under normal physiological conditions, control the tissue damage caused by oxidants [32,33], and there is evidence that oxidant pollutants (as NO_2_, ozone and particles) deplete tissue antioxidant defences causing injury and inflammation, also provoking cell membrane damage and increased membrane permeability [1]. In this sense, otitis is characterized by the inflammation of the mucosal linings of the airy spaces, fact that is essential for the structural and functional restoration of damaged ear tissues [32].

A limitation of this study could be the fact that information on outcomes was collected using a questionnaire rather than clinical records, and the timing of the interview varied between different cohorts (mean 14.5, SD 4.1). Results by cohort showed no significant heterogeneity, but by controlling this limitation, there might have been some recall bias, so we excluded from the analysis those children >30 months in the control visit and adjusted models by child’s age at the control visit.

This study employing the use of a gas cooker as an indoor exposure proxy has the advantage of representing a measure of chronic exposure, while other gas appliances such as gas heaters have the disadvantage of seasonal use patterns. Presence of gas cookers in homes is an indicator of nitrogen dioxide as we saw in INMA previously [34,35] as well as a source of particles (PM_2.5_ and Ultra Fine Particles) and black carbon [36]. Comparing with other longitudinal studies carried out in Europe [37,38] in this multicenter birth cohort study in Spain we found a considerable percentage of individuals exposed to gas cookers, around 45.5%. We tested for different types of gas cooking used by participants (network gas (31.6%) -primarily metane-, or bottle gas (13.9%) - primarily butane and propane in very low concentration-) and results remained stable (data not shown). Nevertheless we have characterized exposure depending upon dichotomous exposure categories (i.e. “presence” or “absence” of the source) and not through other variables than can improve accuracy such as other cooker emission determinants (such as cooking styles and frequency) or the time the child is present in the kitchen when parents are cooking. However, a recent study indicates that the total particle number concentrations in other rooms were comparable to those in the kitchen with a lag of 10 to 12 min [39]. By being aware of this lack of accuracy we interpret that this possible miss-classification bias is non-differential.

Pregnancy and early life are particular susceptible periods for lung development, but the most relevant of these two periods for developing respiratory problems is not clear. We evaluated exposure during pregnancy and to the best of our knowledge no other papers have been published evaluating exposure before birth, although a recent article that also found no association evaluated exposure to gas cooking at birth [38]. In our study, due to the high proportion of homes that maintain the same type of cooking range from pregnancy to the first year (91.2%) (only 3.3% changed from electric to gas and 5.5% changed from gas to electric), it is impossible to distinguish if the relationship found is related only to prenatal exposure or only to postnatal exposure or both. Other limitation in this study is that we have not included postnatal nutrition in the analysis (child intake of fruit and/or vegetable juices and purees).

However, it is more than likely that ongoing efforts by several European multi-centre birth cohort studies such as MeDALL (http://medall-fp7.eu/) or CHICOS (http://www.chicosproject.eu/) that include a large number of study subjects with long follow-up periods during infancy, will cast light on these problems in the future.

## Conclusions

In susceptible subjects (those whose mothers smoke and consumed below average fruit and vegetables) we found an association between exposure to gas cooking during pregnancy and risk of wheezing, chestiness and otitis during the first year of life. But more research is needed regarding not only gas cooking and respiratory health but also the possible effect modifier role of diet and tobacco. In case of be confirmed by other studies, the promotion of protective dietary practices, or supplementation in some cases, as well as healthy habits to avoid tobacco smoke exposure could be an effective prevention strategy to reduce respiratory problems.

## Abbreviations

NO2: Nitrogen dioxide; WHO: World Health Organization; INMA: Envrironment and chilhood study, in Spanish “Infancia y Medio Ambiente”; SD: Standard desviation; LRTI: Lower respiratory tract infection; SGA: Small for gestational age; OR: Odds Ratio; CI: confidence interval; MDA: Malondialdehyde; PM2.5: Particle matter with aerodynamic diameter less than 2.5 μm; MeDALL: Mechanisms of the Development of ALLergy; CHICOS: Developing a child cohort research strategy for Europe.

## Competing interests

All the authors declare no conflicts of interest.

## Authors’ contributions

AE and ME performed the statistical analysis. AE drafted the manuscript. ME and FB helped to draft the manuscript. All authors provided edits and comments to the manuscript. All authors read and approved the final manuscript.

## Supplementary Material

Additional file 1 **Flowchart illustrating the main phases in the study.** Flowchart that show cohort population in differents stages of study classified as ongoing, excluded, miscarriages, foetal deaths, withdrew, lost, etc. Click here for file

Additional file 2 **Differences in child and parental characteristics between participants included and those not included in the present analyses.** Table that show differences between included and excluded population. Click here for file

Additional file 3 **LRTI, wheezing, persistent cough, otitis and chestiness in the first year of life in relation to personal and family characteristics and habits.** Figure that show relation of respiratory outcomes in the first year by individual and family characteristics. Click here for file

Additional file 4 **Adjusted OR (95% CI) of exposure to gas cookers during pregnancy and respiratory problems during the 1st year of life in a birth cohort from selected specific population subgroups.** Figure that show sensitivity analysis. We stratify the analysis by potential effect modifiers as SGA, Preterm and change in the type of cooking. Click here for file
