# Supplementary material for: Prenatal exposure to cooking gas and respiratory health in infants is modified by tobacco smoke exposure and diet in the INMA birth cohort study
Source: Environ Health. 2013 Dec 1;12:100. doi: 10.1186/1476-069X-12-100 (PMC3883519; doi:10.1186/1476-069X-12-100)
Supplement: Additional file 1 — Flowchart illustrating the main phases in the study. Flowchart that show cohort population in differents stages of study classified as ongoing, excluded, miscarriages, foetal deaths, withdrew, lost, etc. [file 1476-069X-12-100-S1.doc]

**Additional file 1. Flowchart illustrating the main phases in the study.**

Pregnant women at 1^st^ trimester of pregnancy

N=2644

Pregnant women at 1^st^ trimester of pregnancy

N=2644

Pregnant women at 3^rd^ trimester of pregnancy N= 2525 (95.5%)

N=…

119 subjects excluded

- 59 miscarriages

- 8 foetal deaths

- 47 withdrew

- 5 lost

Newborns at delivery

N=2506 (94.8%)

19 subjects excluded

- 5 foetal deaths

- 14 withdrew

131 subjects excluded

- 7 died

- 101 withdrew

- 23 lost

Infants at 11-23 months

N=2361 (89.3%)

Subjects with available data for analysis:

- With respiratory outcomes and gas cooking data: N=2235

- After excluding infants who completed questionnaire after 30 months: N=2003

Pregnant women at 3^rd^ trimester of pregnancy N= 2525 (95.5%)

N=…

Newborns at delivery

N=2506 (94.8%)

Infants at 11-23 months

N=2361 (89.3%)

119 subjects excluded

- 59 miscarriages

- 8 foetal deaths

- 47 withdrew

- 5 lost

19 subjects excluded

- 5 foetal deaths

- 14 withdrew

131 subjects excluded

- 7 died

- 101 withdrew

- 23 lost

Subjects with available data for analysis:

- With respiratory outcomes and gas cooking data: N=2235

- After excluding infants who completed questionnaire after 30 months: N=2003
