# Supplementary material for: Prenatal exposure to cooking gas and respiratory health in infants is modified by tobacco smoke exposure and diet in the INMA birth cohort study
Source: Environ Health. 2013 Dec 1;12:100. doi: 10.1186/1476-069X-12-100 (PMC3883519; doi:10.1186/1476-069X-12-100)
Supplement: Additional file 2 — Differences in child and parental characteristics between participants included and those not included in the present analyses. Table that show differences between included and excluded population. [file 1476-069X-12-100-S2.doc]

**Additional file 2.** Differences in child and parental characteristics between participants included and those not included in the present analyses.

|  |  | **Included** | | **Excluded** | |  |
| --- | --- | --- | --- | --- | --- | --- |
|  | | (n= 2003) | | (n= 668 ) | |  |
|  |  | N | (% ) | N | (% ) | p< Chi^2^ |
| Mother’s age | <25 | 129 | 6.44 | 80 | 12.14 | <0.001 |
|  | 25-29 | 649 | 32.42 | 204 | 30.96 |  |
|  | 30-34 | 869 | 43.41 | 245 | 37.18 |  |
|  | 35+ | 355 | 17.73 | 130 | 19.73 |  |
| Country of origin | Spain | 1833 | 91.83 | 569 | 90.46 | 0.210 |
|  | Other | 163 | 8.17 | 60 | 9.54 |  |
| Mother’s educational level | Primary | 468 | 23.40 | 185 | 29.46 | 0.001 |
|  | Secondary | 835 | 41.75 | 254 | 40.45 |  |
|  | University | 697 | 34.85 | 189 | 30.10 |  |
| Social Class | CS I+II | 644 | 32.15 | 180 | 28.57 | <0.001 |
|  | CS III | 518 | 25.86 | 132 | 20.95 |  |
|  | CS IV+V | 841 | 41.99 | 318 | 50.48 |  |
| Working during pregnancy | Not | 531 | 26.55 | 159 | 32.92 | 0.002 |
|  | Yes | 1469 | 73.45 | 324 | 67.08 |  |
| Parity | Neither | 1133 | 56.62 | 340 | 54.05 | 0.194 |
|  | Biparous o multiparous | 868 | 43.38 | 289 | 45.95 |  |
| Smoking during pregnancy | No | 1643 | 82.23 | 379 | 79.12 | 0.075 |
|  | Yes | 355 | 17.77 | 100 | 20.88 |  |
| Parents’ smoking during 1^st^ year | Neither | 1629 | 84.40 | 199 | 80.57 | 0.249 |
|  | One | 199 | 10.31 | 32 | 12.96 |  |
|  | Both | 102 | 5.28 | 16 | 6.48 |  |
| Passive smoking during pregnancy* | Not exposed | 735 | 36.92 | 184 | 38.41 | 0.498 |
|  | Exposed | 1256 | 63.08 | 295 | 61.59 |  |
| Season of Birth | Winter | 532 | 26.56 | 151 | 28.82 | 0.011 |
|  | Spring | 473 | 23.61 | 148 | 28.24 |  |
|  | Summer | 488 | 24.36 | 105 | 20.04 |  |
|  | Autumn | 510 | 25.46 | 120 | 22.90 |  |
| Rural | Non rural | 1890 | 94.69 | 613 | 93.45 | 0.155 |
|  | Rural | 106 | 5.31 | 43 | 6.55 |  |
| Parents allergy history | No | 1152 | 57.57 | 389 | 61.75 | 0.034 |
|  | Yes | 849 | 42.43 | 241 | 38.25 |  |
| Sex | Female | 973 | 48.60 | 264 | 50.19 | 0.466 |
|  | Male | 1029 | 51.40 | 262 | 49.81 |  |
| Preterm (< 37 weeks) | No | 1915 | 96.33 | 477 | 91.38 | <0.001 |
|  | Yes | 73 | 3.67 | 45 | 8.62 |  |
| Low birth weight <2500 gr) | No | 1901 | 95.43 | 479 | 92.47 | 0.001 |
|  | Yes | 91 | 4.57 | 39 | 7.53 |  |
| Breastfeeding (weeks) | 0 | 272 | 13.68 | 72 | 22.43 | <0.001 |
|  | >0-16 | 486 | 24.45 | 98 | 30.53 |  |
|  | 16-24 | 298 | 14.99 | 67 | 20.87 |  |
|  | >24 | 932 | 46.88 | 84 | 26.17 |  |
| Daycare attendance | No | 1330 | 66.80 | 117 | 45.88 | <0.001 |
|  | Yes | 661 | 33.20 | 138 | 54.12 |  |
| Cleaning frequency | <=1 per week | 1132 | 56.57 | 195 | 40.54 | <0.001 |
|  | >1per week | 869 | 43.43 | 286 | 59.46 |  |
| Pets | No | 1474 | 73.92 | 209 | 77.70 | 0.158 |
|  | Yes | 520 | 26.08 | 60 | 22.30 |  |
| Furry pets | No | 435 | 21.78 | 34 | 13.18 | 0.001 |
|  | Yes | 1562 | 78.22 | 224 | 86.82 |  |
| Redecoration | No | 1400 | 69.90 | 151 | 22.60 | <0.001 |
|  | Yes | 603 | 30.10 | 517 | 77.40 |  |
| Damp | No | 1864 | 93.34 | 233 | 90.31 | 0.051 |
|  | Yes | 133 | 6.66 | 25 | 9.69 |  |
| Ventilation | >2 h per day | 1249 | 62.58 | 316 | 66.25 | 0.098 |
|  | <=2 h per day | 747 | 37.42 | 161 | 33.75 |  |
| Air conditioning | No | 1401 | 69.95 | 409 | 84.85 | <0.001 |
|  | Yes | 602 | 30.05 | 73 | 15.15 |  |
|  |  |  |  |  |  |  |
| Child’s age (months) at visit |  | Mean | SD | Mean | SD |  |
|  | All | 14.5 | 4.1 | 28.1 | 9.9 | <0.001 |
|  | Asturias | 24.8 | 2.9 | 33.2 | 5.8 |  |
|  | Gipuzkoa | 13.8 | 1.2 | 13.8 | 1.3 |  |
|  | Sabadell | 14.1 | 0.8 | 14.3 | 0.9 |  |
|  | Valencia | 12.0 | 1.1 | 11.8 | 0.4 |  |
| *Home, restaurants and other homes exposure. SD (Standard Deviation); P (percentile) | | | | | | |
