# Supplementary material for: Prenatal exposure to cooking gas and respiratory health in infants is modified by tobacco smoke exposure and diet in the INMA birth cohort study
Source: Environ Health. 2013 Dec 1;12:100. doi: 10.1186/1476-069X-12-100 (PMC3883519; doi:10.1186/1476-069X-12-100)

**Additional file 3**: LRTI, wheezing, persistent cough, otitis and chestiness in the first year of life in relation to personal and family characteristics and habits.


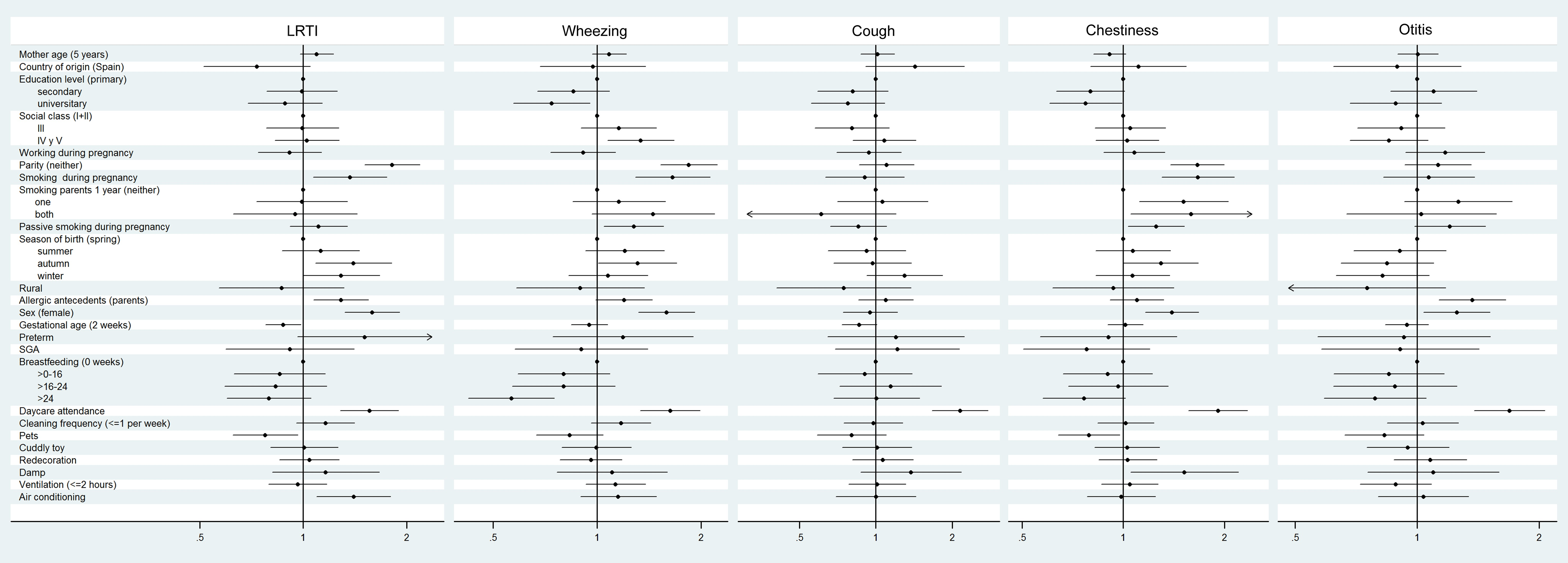

Supplement: Additional file 3 — LRTI, wheezing, persistent cough, otitis and chestiness in the first year of life in relation to personal and family characteristics and habits. Figure that show relation of respiratory outcomes in the first year by individual and family characteristics. [file 1476-069X-12-100-S3.doc]
