# Supplementary material for: Prenatal exposure to cooking gas and respiratory health in infants is modified by tobacco smoke exposure and diet in the INMA birth cohort study
Source: Environ Health. 2013 Dec 1;12:100. doi: 10.1186/1476-069X-12-100 (PMC3883519; doi:10.1186/1476-069X-12-100)

**Additional file 4**: Adjusted OR (95% CI) of exposure to gas cookers during pregnancy and respiratory problems during the 1st year of life in a birth cohort from selected specific population subgroups.


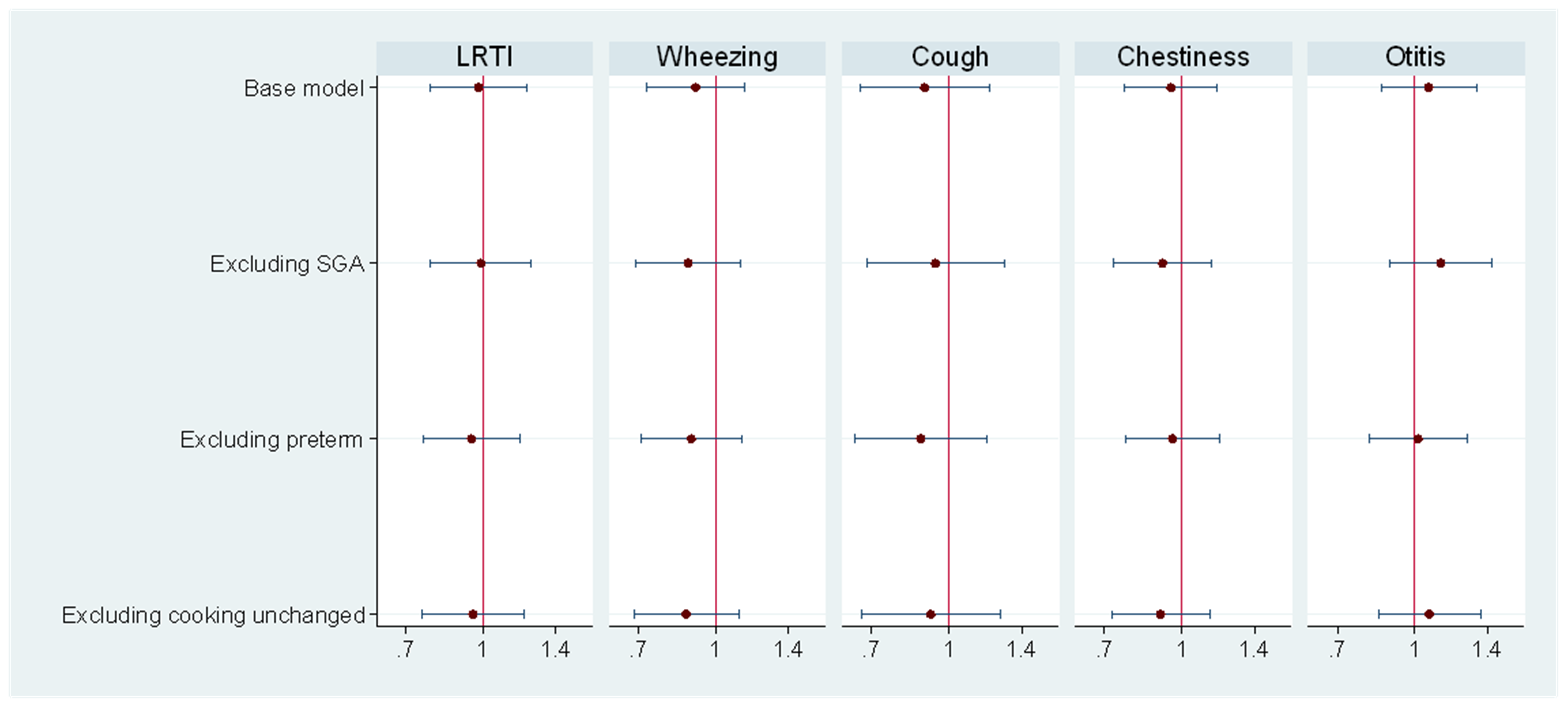

Supplement: Additional file 4 — Adjusted OR (95% CI) of exposure to gas cookers during pregnancy and respiratory problems during the 1st year of life in a birth cohort from selected specific population subgroups. Figure that show sensitivity analysis. We stratify the analysis by potential effect modifiers as SGA, Preterm and change in the type of cooking. [file 1476-069X-12-100-S4.doc]
